# Hypoxia-induced down-regulation of microRNA-449a/b impairs control over targeted SERPINE1 (PAI-1) mRNA - a mechanism involved in SERPINE1 (PAI-1) overexpression

**DOI:** 10.1186/1479-5876-9-24

**Published:** 2011-03-04

**Authors:** Michaela Muth, Kais Hussein, Christoph Jacobi, Hans Kreipe, Oliver Bock

**Affiliations:** 1 Institute of Pathology, Hannover Medical School, Carl-Neuberg-Strasse 1, 30625 Hannover, Germany; 2 Department of Pediatric Nephrology, Hannover Medical School, Carl-Neuberg-Strasse 1, 30625 Hannover, Germany

## Abstract

After publication of our article [1], we realized the need for posting a correction note in order to prevent i) overinterpretation of some results by the readers and ii) concerns about potentially unintended misguidance by the authors.

## Correction

Array-based gene expression analysis (mRNA, miRNA, respectively) was performed in 2 independent experiments resulting in 2 values for differentially expressed targets (Figures one and three). The resulting error bars therefore appear symmetrical.

**Figure 1:**
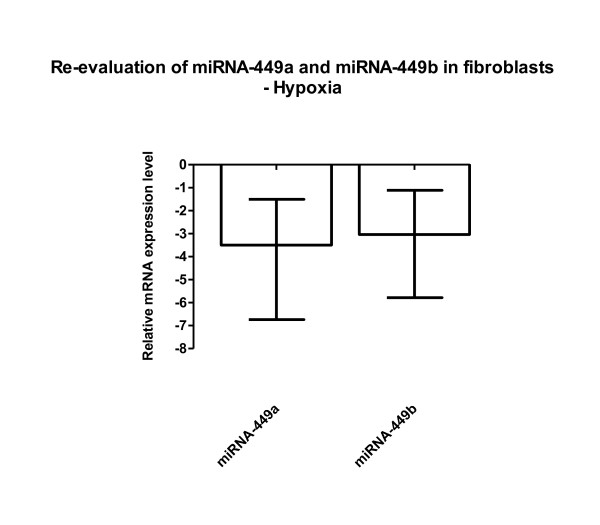
**Re-evaluation of miRNA-449a/b in fibroblasts (original Figure 2)**. Three independent experiments confirmed down-regulation of miRNA-449a/b under hypoxia. All calculations were performed relative to RNU48 in the cell line F-18. Mean, minimum and maximum values are shown.

**Figure 2:**
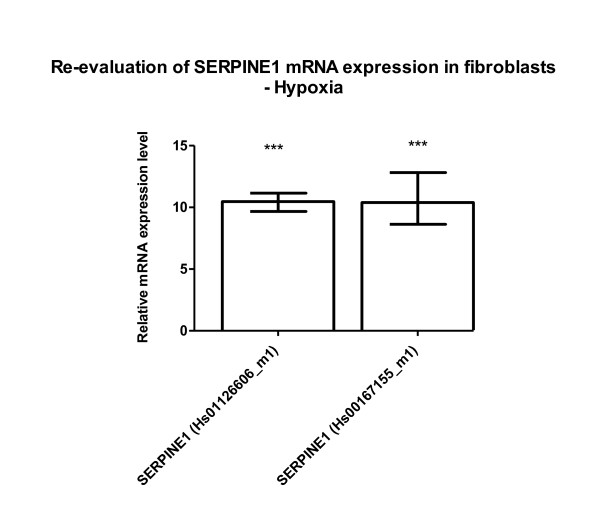
**Re-evaluation of SERPINE1 mRNA expression in fibroblasts under hypoxia (original Figure 4)**. Two different gene expression assays were applied to confirm SERPINE1 overexpression relative to POLR2A in fibroblasts cultured under hypoxia compared with fibroblasts cultured under normal oxygen conditions. Mean, minimum and maximum values are shown from three independent experiments reproduced in the cell line F-18 showing a much stronger induction than illustrated in the initial figure 4 (*** p < 0.001).

**Figure 3:**
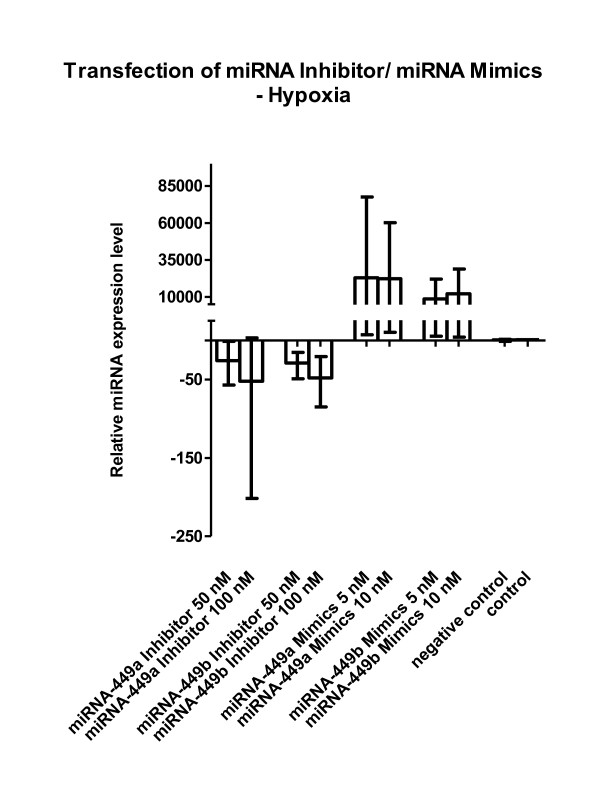
**Transfection of miRNA-449a/b inhibitors and mimics and effects under hypoxia (original Figure 5)**. Primary human fibroblasts were transfected with miRNA-449a/b inhibitor or miRNA-449a/b mimics and were cultured under hypoxia. Inhibition strongly decreased miRNA-449a/b expression. Mimics showed an exaggerated effect on the miRNA-449a/b level. The mean, minimum and maximum of calculations relative to reference gene RNU48 compared with non-transfected cells (= control) and cells transfected with negative control siRNA only (= negative control) cultured under hypoxia are depicted. Results from 3 independent experiments are shown for cell line F-18, but were likewise demonstrable in M15D.

**Figure 4:**
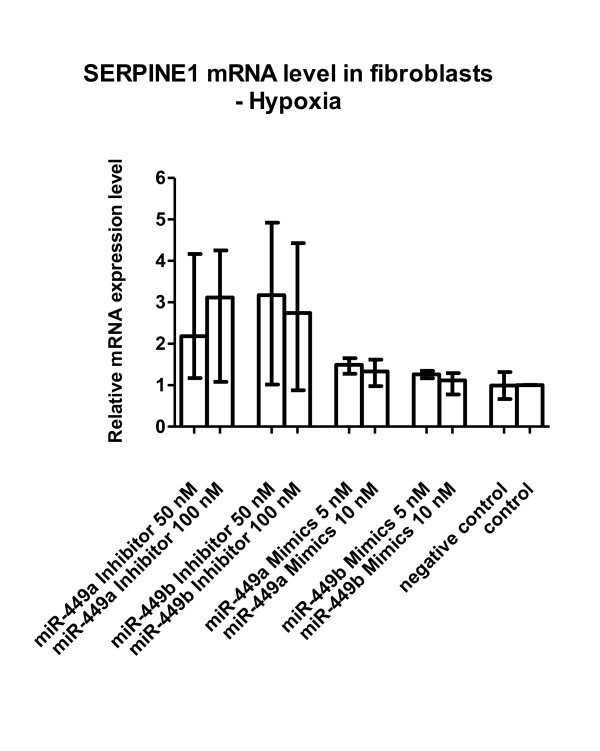
**SERPINE1 mRNA level in fibroblasts under hypoxia (Figure 6)**. Transfection of miRNA-449a/b inhibitors additionally increased the hypoxia-induced SERPINE1 mRNA expression. The mean, minimum and maximum of calculations relative to reference gene POLR2A compared with non-transfected cells (= control) or cells transfected with negative control siRNA only (= negative control) cultured under hypoxia in 3 independent experiments are depicted. The miRNA-449 mimics showed virtually no down-regulation of SERPINE1 mRNA level.

In the published figures two, four, five and six, data from 1 experiment, i.e. 2 measurements, are shown, likewise resulting in symmetrical error bars. These figures are based on data sets underlying version 1 of the submitted manuscript. Version 2 of the manuscript for publication should contain figures two, four, five, and six (figures 1,2,3, and 4 in this correction) and the underlying data sets from 3 independent experiments with 2 measurements (values) per experiment. *Unfortunately, internal errors eventually have led to discrepancies between intended illustration of data sets and the published ones.*

We have now incorporated the valid figures and figure legends. The underlying data set is shown in the supplement of this correction note (additional file 1). The corrected figures five and six (figures 3 and 4 here) now need particular attention because a subtle interpretation is necessary.

When we transfected either miRNA-449a/b inhibitors or mimics, we noted the expected decrease or increase of the miRNA-449 subtype under investigation (Figure five) (figure 3 here), an effect only investigated under hypoxic culture conditions. The constitutive low miRNA-449a/b levels in fibroblasts *in vitro *(threshold cycles in real-time PCR experiments >32) which further decreased by hypoxia (as shown in Figure two) (figure 1 here) may be an explanation why miRNA-449a/b mimics have had such an exaggerated effect. The broad range of expression following transfection of miRNA inhibitors and miRNA mimics in the 3 experiments abrogated the statistical significance shown in the initial figure five.

Figure six (figure 4 here) needed a substantial revision to allow a clear-cut interpretation of our findings. As stated in the results section, hypoxia per se induces i) down-regulation of miRNA-449a/b and also miRNA-518a-3p (Figure one) and ii) along with other genes up-regulation of SERPINE1 (PAI-1) (Figure three). These findings are totally independent of any transfection approaches with miRNA species such as miRNA-449a/b. When hypoxic fibroblasts were transfected with miRNA-449a/b inhibitors, SERPINE1 (PAI-1) mRNA was additionally increased up to 3-fold while miRNA-449a/b mimics showed no effect with the concentrations used. The "stumbling block" in the original figure six was our aim to illustrate the transfection-independent effect of hypoxia-induced SERPINE1 (PAI-1) mRNA expression by incorporating a dotted line. The line was intended to highlight the additional increase of SERPINE1 (PAI-1) mRNA by miRNA-449a/b inhibitors. Unfortunately, it could also suggest a false-positive effect of miRNA-449a/b mimics, i.e. notable down-regulation of SERPINE1 (PAI-1) mRNA. It must be emphasized that the latter effect is not demonstrable because the negative control showed almost identical SERPINE1 (PAI-1) mRNA levels. We therefore revised Figure six (figure 4 here) which now shows the apparent effect of miRNA-449a/b inhibitors but not of the mimics. We meanwhile resigned from insisting on a statistically robust effect of miRNA-449a/b inhibitors on PAI-1 mRNA expression. Dependent on the statistical test used we were able to demonstrate a tendency only for the miRNA-449b inhibitor at 50 nM (p < 0.05).

The biological significance of the hypoxia-dependent effect on miRNA-449a/b levels demonstrable in vitro remains to be further investigated in vivo. As shown for organ fibrosis by using kidney allograft remodelling (Figure eight), SERPINE1 (PAI-1) mRNA was increased whilst miRNA-449a/b species were decreased. We do not know the extent of the miRNA-449a/b contribution to SERPINE1 (PAI-1) mRNA level in hypoxic environments in remodelled tissues. However, we believe that, based on the in vitro data shown, a subtle mechanism contributes to higher SERPINE1 (PAI-1) mRNA levels through down-regulated miRNA-449a/b.

Until we were given the opportunity to prepare this correction note, we reproduced the hypoxic cell culture experiments in a standardized fashion. Our lab was recently equipped with a Hypoxystation H35 from DonWhitley and instead of using the "Anaeropack for cell culture" (which, by definition, produces an almost anoxic condition without monitoring the definite concentration), we now culture cells under controlled and monitored oxygen concentrations.

Because of the continuously adjustable oxygen concentration in the hypoxystation, we found that 5 percent oxygen or less is needed to induce a decrease of miRNA-449a/b expression levels in different cell lines. Interestingly, oxygen concentrations of 1 percent decreased the miRNA-449a/b level more than 5 percent did. In contrast, SERPINE1 (PAI-1) mRNA apparently needs oxygen levels below 1 percent to show a remarkable up-regulation. Analyses were not restricted to fibroblasts but also included leukemic and solid tumor cell lines. Since this correction note is not a platform for presentation of new data, we only wish to underline our still ongoing activities in this field.

We offer our sincere apologies to the scientific community for any confusion or misleading interpretation we may have caused.

## Authors' Note

Katharina Theophile, the 2^nd ^author in the author list for the original article, is omitted from the authorship list on this correction article as the remaining authors have not been able to contact her to confirm her approval of this correction article.

## Supplementary Material

Additional file 1 Data underlying corrected Figures 2, 4, 5, 6. Click here for file
