# Supplementary material for: Hypoxia-induced down-regulation of microRNA-449a/b impairs control over targeted SERPINE1 (PAI-1) mRNA - a mechanism involved in SERPINE1 (PAI-1) overexpression
Source: J Transl Med. 2011 Mar 4;9:24. doi: 10.1186/1479-5876-9-24 (PMC3066127; doi:10.1186/1479-5876-9-24)
Supplement: Additional file 1 — Data underlying corrected Figures 2, 4, 5, 6. [file 1479-5876-9-24-S1.DOC]

|  | miRNA-449a | miRNA-449b |
| --- | --- | --- |
| Mean | - 3.5 | - 3.0 |
| Minimum | - 6.7 | - 5.8 |
| Maximum | - 1.5 | - 1.1 |

**Additional file 1: Data underlying corrected Figures 2, 4, 5, 6**

**Figure 2**

**Figure 4**

|  | SERPINE1  (Hs01126606_m1) | SERPINE1  (Hs00167155_m1) |
| --- | --- | --- |
| Mean | 10.5 | 10.4 |
| Minimum | 9.7 | 8.6 |
| Maximum | 11.2 | 12.8 |

**Figure 5**

|  | -449a Inhibition | | -449b Inhibition | | -449a Mimics | | -449b Mimics | | Negative | Control |
| --- | --- | --- | --- | --- | --- | --- | --- | --- | --- | --- |
|  | 50nM | 100nM | 50nM | 100nM | 5nM | 10nM | 5nM | 10nM |
| Mean | - 25.7 | - 52.1 | -28.8 | -47.9 | 22988 | 22282 | 8721 | 12116 | 0.7 | 1.02 |
| Minimum | - 57.0 | -201.6 | -49.1 | -84.9 | 7.3 | 10.3 | 5.4 | 4.2 | -1.1 | 1.01 |
| Maximum | - 1.1 | 3.2 | -15.1 | -20.6 | 77588 | 60178 | 22020 | 28908 | 1.3 | 1.04 |

**Figure 6**

|  | -449a Inhibition | | -449b Inhibition | | -449a Mimics | | -449b Mimics | | Negative | Control |
| --- | --- | --- | --- | --- | --- | --- | --- | --- | --- | --- |
|  | 50nM | 100nM | 50nM | 100nM | 5nM | 10nM | 5nM | 10nM |
| Mean | 2.2 | 3.1 | 3.2 | 2.7 | 1.5 | 1.3 | 1.3 | 1.1 | 1.0 | 1.00 |
| Minimum | 1.2 | 1.1 | 1.0 | 0.9 | 1.3 | 1.0 | 1.2 | 0.8 | 0.7 | 1.00 |
| Maximum | 4.2 | 4.3 | 4.9 | 4.4 | 1.7 | 1.6 | 1.4 | 1.3 | 1.3 | 1.01 |

Three independent experiments were performed with 2 measurements (values) per experiment, resulting in 1 mean per experiment. The 3 means from the 3 experiments were then taken for statistics and figure preparation.
